# Study of Separation and Fouling of Reverse Osmosis Membranes during Model Hydrolysate Solution Filtration

**DOI:** 10.3390/membranes7040068

**Published:** 2017-12-15

**Authors:** Olumoye Ajao, Mohamed Rahni, Mariya Marinova, Hassan Chadjaa, Oumarou Savadogo

**Affiliations:** 1 Research Unit on Energy Efficiency and Sustainable Development of the Forest Biorefinery, Chemical Engineering Department, Polytechnique Montreal, C.P. 6079 succ. Centre-Ville, Montréal, QC H3C 3A7, Canada; mariya.marinova.1@ulaval.ca (M.M.); oumarou.savadogo@polytmtl.ca (O.S.); 2 Centre National en Électrochimie et en Technologies Environnementales, Shawinigan, 2263, Avenue du Collège, Shawinigan, QC G9N 6V, Canada; mrahni@cnete.qc.ca (M.R.); hchadjaa@cnete.qc.ca (H.C.)

**Keywords:** prehydrolysate, reverse osmosis, filtration, furfural production, hemicelluloses biorefinery, Taguchi experimental design

## Abstract

Prehydrolysate, a dilute solution consisting mainly of pentoses, hexoses, and lesser quantities of organic acids, furfural and phenolics, is generated in the Kraft dissolving pulp process. An obstacle facing the valorization of the solution in hemicellulose biorefineries, by conversion of the sugars into bioproducts such as furfural, is the low sugar concentration. Membrane filtration is typically proposed in several hemicellulose based biorefineries for concentrating the solution, although they are usually generated using different wood species, pretreatment methods, and operating conditions. However, the chemical composition of the solutions is generally not considered. Also, the combined effect of composition and operating conditions is rarely investigated for biorefinery applications. The purpose of this work was to determine the impact of the prehydrolysate composition and operating parameters on the component separation and permeate flux during membrane filtration. Using model prehydrolysate solutions, two commercial reverse osmosis (RO) membranes were screened, and one was selected for use, based on its higher sugar and acetic acid retention. A Taguchi L18 experimental design array was then applied to determine the dominant parameters and limiting factors. Results showed that the feed pressure and temperature have the highest impact on permeate flux, but the least effect on sugar retention. Further experiments to quantify flux decline, due to fouling and osmotic pressure, showed that furfural has the highest membrane fouling tendency, and can limit the lifetime of the membrane. Regeneration of the membrane by cleaning with a sodium hydroxide solution is also effective for reversing fouling. It has been demonstrated that RO can efficiently and sustainably concentrate wood prehydrolysate.

## 1. Introduction

**Figure 1:**
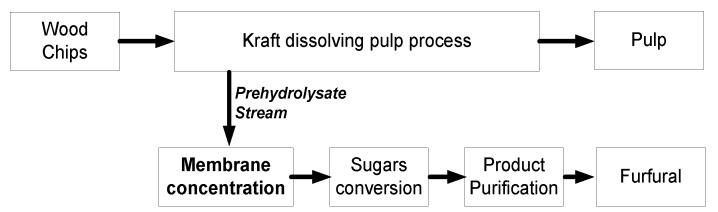
Schematic representation of an Integrated Forest Biorefinery for furfural production.

Since the closure of several Kraft pulp mills in the past decade in Canada, efforts to reposition the sector has resulted in a revision of the business model of presently operating mills by transforming them into Integrated Forest Biorefineries (IFBRs) [1]. An IFBR generally involves the diversification of the product portfolio of a mill by integrating new processes for sustainable products, such as biochemicals, bioenergy, biofuels, or biomaterials. Other advantages of a biorefinery integrated into a Kraft dissolving pulping mill include: (i) the existing infrastructure on site can reduce the investment costs for the biorefinery; (ii) skilled manpower with experience in biomass handling and processing is available on site; (iii) the heating and cooling utility requirements can be provided (partially or totally) by the mill. Five Kraft pulp mills in Canada have been converted from paper grade to dissolving grade pulp processes in the past. In 2014, three other mills were under conversion, due to an increase in the price and global demand for dissolving grade pulp, which can be used for the manufacture of textile fibers [2]. Dissolving Kraft pulp mills are suitable receptors of a sugar platform biorefinery, because the prehydrolysis of the wood chips to remove the hemicellulose fraction is carried out prior to cooking, thus making hemicellulosic sugars available for new products. Presently, the hemicelluloses are typically combusted to produce energy in the chemical recovery cycle of the pulping process. Several methods exist for the prehydrolysis of wood chips. The use of hot water is advantageous, because it is a mature, cost-efficient technique, and does not require the use of chemicals. Furthermore, the hemicellulose sugars can be easily extracted and recovered. The resulting stream is dilute, and contains a mixture of pentose and hexose sugars with less than 4% *w*/*v* total sugar [3], and small quantities of organic acids and phenolics. Valorization of this stream via a biochemical pathway to produce biofuels, such as ethanol and butanol, or a chemical pathway for bioproducts, such as furfural or xylitol, is possible. In mills with a hardwood feedstock, it is advantageous to produce a platform bio-product, such as furfural, because the pentoses, which make up the highest proportion of the prehydrolysate stream, are more difficult to ferment into biofuels than hexoses. Also, the production cost of biofuels from such a stream is higher than from alternative feedstock, like sugar cane or corn [4]. Furfural is a platform chemical, which can replace several industrial organic compounds that are presently produced from crude oil. An exhaustive overview of furfural feedstock, production pathways, derivatives and applications, energy intensity, and the design of a cost-efficient process for its production, was previously presented [5,6]. To produce high purity furfural from prehydrolysate in an IFBR, three main steps are required: (i) concentration of the generated prehydrolysate; (ii) sugars (pentose) conversion by a dehydration reaction into furfural; and (iii) product purification by distillation as shown in Figure 1.

The concentration step is specific to an IFBR based on Kraft pulp mill for dissolving pulp production, and it is essential for reducing the energy cost and process equipment dimension. Furthermore, a low pH is required to catalyze the conversion of sugars into furfural. Organic acids, such as formic [7] and acetic acids, are suitable catalysts that can also reduce the mineral acid requirement. It is therefore important that the method for concentration retains the organic acids present in the prehydrolysate along with the sugars. Multiple-effect evaporators are efficient and widely used in industrial applications for concentration when large volumes of water must be recovered, but two drawbacks are associated with its potential use in the furfural biorefinery. Firstly, a large amount of energy in the form of steam is required, and acetic acid, the main organic acid, will be lost because of its volatility. These drawbacks can be avoided by membrane filtration, an energy efficient technique for concentration and water recovery in a wide range of industrial applications. Several studies have been conducted on membrane performance during saccharide filtration by the Mänttäri research group [8,9,10,11], and also the recovery of hemicelluloses from pulp mills by the research group of A-S Jönsson [12,13,14]. In general, their investigations covered hemicellulose purification, filtration performance, flux increase, and decreased fouling. However, there was no direct link to requirements for further conversion in a biorefinery process. Other researchers have treated membrane application in biorefineries with emphasis on the removal of inhibitors from real and synthetic hydrolysate solutions prior to the conversion of sugars into biofuels or biochemicals by fermentation [15,16,17,18,19,20]. Some of those earlier reported studies have been reviewed [15,16,17,18,19,20,21,22,23,24,25,26,27,28,29,30,31,32], and a few relevant examples are summarized in Table 1.

Industrial applicability of membrane filtration, coupled with a pulping process, has also been investigated at the pilot scale [37,38]. Except for the related work by the authors [28], no other study on the application of either reverse osmosis or nanofiltration membranes for hemicellulose concentration in a furfural process was found in the existing literature. Previous work of the authors has shown that flux decline could limit the application of membrane filtration during prehydrolysate concentration. However, the study did not reveal the cumulative impact and respective contributions of the main prehydrolysate components on the flux and component retention characteristics. This was because a real prehydrolysate solution with a fixed chemical composition was used in the reported experiments. Furthermore, no attempt has been made to simultaneously concentrate the sugars and other compounds. The objectives of this work are: (i) to expand knowledge on how the interaction between an organic membrane and the chemical compounds in a prehydrolysate solution can impact membrane application when several components are to be retained; and (ii) to provide a basis for the evaluation of the technical and economic feasibility of membrane applications in biorefineries for furfural production. Synthetic solutions representing the composition of prehydrolysate from dissolving pulp mills were used. The experiments were carried out in five phases: (I) to identify membranes with suitable component retention; (II) to determine the optimum conditions for high permeate flux; (III) to evaluate the impact of prehydrolysate composition and feed conditions on flux decline; (IV) to assess the extent to which results from related experiments are applicable; (V) to elucidate the membrane retention when single- and mixed-solute solutions are concentrated.

The Taguchi design of experiments method has been successfully applied to gather information on main and interaction effects of design parameters from a minimal number of experiments [39], and was employed in this study. The steps of the Taguchi method are discussed in Section 3.1.

## 2. Materials and Methods

### 2.1. Membranes

Two similar spiral wound commercial RO membranes, Dow Filmtec TW30-2540 and Dow Filmtec BW30-4040, were used in this study. They were made of polyamide thin film composites, had a continuous operation pH range of 2–11, maximum operating temperature of 45 °C, maximum operating pressure of 4100 kPa and molecular weight cut off (MWCO) of about 100 Da. The membranes were cut lengthwise and opened up. They were immersed in a solution of 1% *w*/*v* of sodium metabisulfite to loosen the membrane pores and prevent the growth of microorganisms. Prior to the filtration experiments, flat sheets were cut from the membrane roll, and placed in distilled water for at least three days to remove the sodium metabisulfite and condition the membrane. 

### 2.2. Experimental Setup

**Figure 2:**
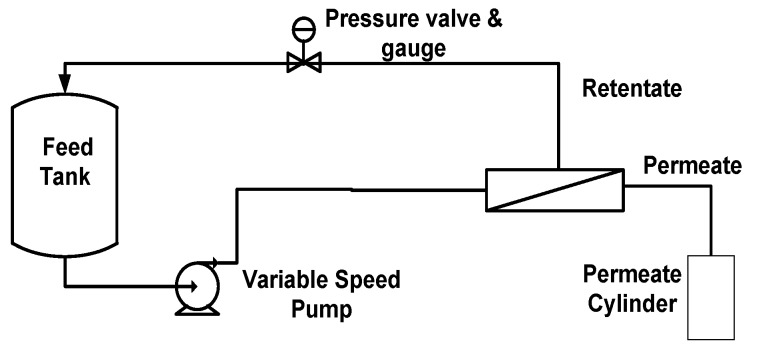
Schematic of the reverse osmosis concentration setup in batch mode.

A lab-scale SEPA CF II (GE Osmonics, Minnetonka, MN, USA) cross-flow flat-sheet membrane test unit was used in this experimental study. The schematic of the experimental setup is shown in Figure 2. It had a rectangular tangential flow canal that can accommodate any type of flat-sheet membrane with the following dimensions: 9.6 cm (breadth), 14.5 cm (length), and 0.1 cm (height). A hydraulic hand pump (SPX, maximum pressure of 7000 kPa) was used to pressurize the flat sheet between the two stainless steel half cells. The feed tank is made of stainless steel, and it had a capacity of 4 liters. It had a hollow wall with glycol circulated between the walls to control the temperature of the model solution in the tank. A Hydra Cell M03 type high-pressure pump (11.25 L/min maximum volume flow delivery) is used to feed the solution to the membrane cell.

In a batch run for concentration, the permeate stream is collected in a cylinder while it is directed back into the feed tank in a closed loop. A closed loop makes it possible to evaluate the membrane separation without any interference from the concentration of the model solution.

### 2.3. Model Solution Preparation

All chemicals were reagent grade and obtained from different suppliers. d-Xylose was obtained from Bioshop Canada, d-glucose and acetic acid were obtained from Fisher Scientific, while furfural and syringaldehyde were supplied by Sigma Aldrich. They were used as received without any further purification. The main physicochemical properties of the compounds are summarized in Table 2.

Model solutions for each experimental run were prepared by dissolving predetermined amounts of glucose, xylose, syringaldehyde, acetic acid, and furfural in distilled water. The mixture was then heated to 40 °C, and thoroughly mixed with magnetic stirrers, before allowing it to cool to the test temperature level. All final solutions contained 35 g/L of glucose and 10 g/L of xylose, which are typical sugar compositions of prehydrolysate generated by hot water treatment of hardwood chips. The compositions of the other chemical compounds were varied to assess their effects on filtration performance. The pH of the solutions was not adjusted.

### 2.4. Prehydrolysate Solution

The prehydrolysate used for comparison with the model solution in this study was generated using previously described wood furnish, equipment, and operating conditions [28]. A liquor to wood ratio of 3:1 was applied, and the concentrations of the key components in the prehydrolysate solutions were as follows: total sugars (21 g/L), acetic acid (3.8 g/L), phenolic compounds (4.7 g/L), furfural (0.7 g/L), and hydroxymethylfurfural (0.09 g/L). Monomeric sugars made up 16% of the total sugars, while the ratio of pentoses to hexoses was 4:1. 

### 2.5. Filtration Procedure

Preliminary characterization tests were carried out on a virgin membrane to determine the pure water permeate flux and its evolution over extended periods of use. This served as a benchmark for all the membranes used in the concentration experiments. Prior to concentration runs, the two membranes (TW30 and BW30) were screened under identical conditions, to select the one with the most suitable separation characteristics for the 18 concentration runs. A fresh membrane sheet was used in all experimental runs. In the screening run, 1.5 L of model solution was supplied into the feed tank, and continuously filtered in a closed loop run (the permeate stream was directed back into the feed tank). Approximately 10 mL was collected at 15 min intervals for a total of 90 min, from the permeate and retentate streams. The permeate flow rate was also measured at the same interval. During the batch concentration operation mode (Figure 2), 1.6 L of model solution was introduced into the feed tank, and the permeate was collected in a cylinder. Approximately 10 mL of permeate sample was taken after each 200 mL of withdrawn permeate. To have a sugar concentration factor of 3, a total of 1.06 L of permeate was withdrawn. Samples of the model solution in the feed tank were taken before and after concentration. All the collected samples were analyzed for concentrations of glucose, xylose, acetic acid, furfural, and syringaldehyde. Before and after filtration of the model solutions, the permeate flow (L/min) at 690, 1378, and 2068 kPa was determined using distilled water at a constant cross-flow velocity of 0.4 m/s, in order to determine the fouling of the membrane.

### 2.6. Membrane Cleaning Procedure

Cleaning tests after filtration were carried out by using deionized water, which had the pH adjusted to 12 by adding a 19 M NaOH solution. About 3 L of the solution was fed into the tank. The filtration system was operated at room temperature (32 ± 2 °C) and atmospheric pressure (101 kPa) for 30 min in a closed loop. Subsequently, the system was drained and rinsed with 8 L of deionized water before pure water flux measurements were made.

### 2.7. Analytical Methods

The concentrations of xylose and glucose were quantified by HPLC (Agilent Technologies, Waldbronn, Germany) equipped with a refractive index (RI) detector and Inertsil NH2 (250 × 4.6 mm) column. An acetonitrile/water mixture (CH_3_CN 80%/H_2_O 20%) was used as eluent. The flow rate of the mobile phase was 2 mL/min, and the column temperature was 40 °C. Furfural concentration was analyzed by the same HPLC using a 280 nm diode array detector (DAD) and a Nucleosil C18 (150 × 4.6 mm) column. A mixture of acetonitrile/water/acetic acid (CH_3_CN 15%/H_2_O 84%/C_2_H_4_O_2_) was utilized as eluent. The flow rate of the mobile phase was 1 mL/min, and the column temperature was 25 °C. Acetic acid was analyzed using a 210 nm DAD coupled with an Inertsil ODS-3 (150 × 4.6 mm) column. The mobile phase was a mixture of 50 mM potassium phosphate, adjusted to a pH of 2.8 with phosphoric acid (H_3_PO_4_) and acetonitrile (KH_2_PO_4_ 99%-CH_3_CN 1%), and fed at 1.25 L/min into the column at 40 °C. 

Syringaldehyde quantification was done by colorimetric analysis using Folin–Ciocalteau reagent, in a procedure similar to that described by Singleton and Rossi [44]. In each tube, 500 µL of diluted model solution sample was added followed by 3.8 mL of water and 200 µL of Folin–Ciocalteau reagent. After 3 min, 500 µL of sodium hydroxide (6%, *w*/*v*) was added into the tube, and the tube allowed to stand at room temperature in the dark. After 1 hour, the absorbance (725 nm) was measured with a visible Novaspec II spectrophotometer (Pharma Biotech, Cambridge, UK). A calibration curve was prepared, using a standard solution of gallic acid (50, 100, and 150 mg/L). Results were expressed as mg gallic acid equivalents (GAE)/L of syringaldehyde.

The pH and conductivity of the feed, permeate, and concentrate streams were determined by an Accumet AB250 pH/ISE Meter (Fisher Scientific, Hampton, NH, USA) and a Orion 3-Star Benchtop Conductivity Meter (Thermo Scientific, Waltham, MA, USA).

## 3. Theory and Computation Method

### 3.1. Design of Experiments (DOE) by the Taguchi Method

The Taguchi method is a simple, systematic, and efficient approach to determine the factor levels that will result in the best performance of a process. It requires the use of special arrays (standard orthogonal arrays) that are derived from the degree of freedom of the process parameters [45]. In this case, the method was adapted for diagnosing the reasons of non-optimal performance of the membrane.

The steps for carrying out a Taguchi experiment design are: (i) experiment planning; (ii) conducting the experiments; and (iii) results analysis and evaluation [46,47]. Experiment planning consists of defining the variables, selecting their levels, and using standard orthogonal arrays to determine the number of experiments. After the experiments are conducted, an analysis of variance (ANOVA) is carried out. This is followed by determining the optimum combination of variables, and confirmatory experiments to validate the predictions obtained by the analysis of variance. The Taguchi method involves a data transformation for analysis of the experimental data, the variation of the measured responses is expressed as the signal-to-noise ratio (S/N) [48]. Three different standard types of S/N ratios can be used depending on the desired objective, they are characterized as:

- Smaller is better (S/N)_S_, to minimize the measured response;
- Nominal is the best (S/N)_N_, to attain a set value for the measured response;
- Larger is better (S/N)_L_, to maximize the measured response.

For 6 parameters having 3 levels each, 18 experiment runs were required with the adopted L18 orthogonal array. The six controlling factors were: A (furfural concentration), B (acetic acid concentration), C (phenolic concentration), D (temperature), E (pressure), F (cross-flow velocity). Their levels are given in Table 3. All the solutions contained a fixed amount of sugars, 35 g/L of xylose, and 10 g/L of glucose.

The lower and upper bounds for the levels show the range of possible values. The midpoints for the concentrations (A, B, and C) reflect the typical concentrations to be expected, while the midpoints for the operating parameters (D, E, and F) represent their center points.

The experimental run matrix was designed using the statistically analytical software package STATISTICA 10 (StatSoft software, Tulsa, OK, USA), and is shown in Table 4. The same software was used for the interpretation of the results.

### 3.2. Measures of Membrane Perfomance

The volumetric concentration factor (VCF) achieved with a membrane can be defined by Equation (1), where V represents the volume. In a closed loop run with no concentration, such as the membrane screening experiments, V_feed_ = V_concentrate_.

VCF = V_feed_/V_concentrate_ (1)

The permeate flux J, during each experimental run, was estimated from the volume of permeate V, that flows through a specific surface area of the membrane A, over a period of time t, as given in Equation (2).

J = V/(A.t)
(2)

For a two component system, it is assumed that the solution diffusion model is a valid means of relating the flux with the operating and osmotic pressure of the membrane [49]. This simplified relationship, as presented in Equation (3), was used to estimate the osmotic pressure from the experimentally determined permeate flux and pressure. ∆P is the transmembrane pressure, and ∆π the osmotic pressure difference between the feed and permeate side, and A_w_ the solute transport coefficient.

J_v_ = A_w_ (∆P − ∆π) (3)

To assess the separation performance of the membranes at different conditions, the percentage retention of all the components in the fed model solution was calculated from Equation (4).

(4) $$\%\;R_{i}={\left(1-\frac{C_{\mathrm{permeate}}}{C_{\mathrm{feed}}}\right)}_{i}\times 100$$

where the C_permeate_ is the concentration of the *i*th component in the permeate and C_feed_ its concentration in the feed. The recovery of the *i*th component was validated by using the mass balance equation given in Equation (5).

C_feed_V_feed_= C_permeate_V_permeate_ + C_concentrate_V_concentrate_ (5)

During membrane operation, reduction of the permeate flux over time occurs. This can be due to any or a combination of increased osmotic pressure, compaction of the membrane, membrane fouling, or concentration polarization. As the solutes become more concentrated, the osmotic pressure, a function of solute concentration, also increases. A higher feed pressure is required to compensate for this increased osmotic pressure (Equation (2)). Compaction is a deformation of the membrane as a result of physical compression when it is placed under pressure, and it could either be reversible or irreversible [50]. It leads to a decline of the permeate flux, and can also alter the separation efficiency of a membrane. However, compensation for compaction can be made at the design stage of a membrane system. Concentration polarization is a phenomenon that arises when the solute concentration near the surface of the membrane exceeds the concentration in the bulk liquid, resulting in lower transmembrane flow [51]. Fouling is a resistance to mass transfer leading to a reduction in the permeate flux of a membrane. It occurs as a consequence of the deposition and accumulation of materials on the surface during operation. The main types of fouling are classified in four groups [52]:

- organic fouling from organic compounds
- particulate fouling due to the presence of suspended and colloidal matter
- biofouling due to the growth of bacteria after their adhesion to the membrane surface
- scaling due to the precipitation of compounds that are sparingly soluble.

Fouling is inevitable and cannot be completely eliminated, but it can be reduced [53].

## 4. Results and Discussion

### 4.1. Membrane Screening

**Figure 3:**
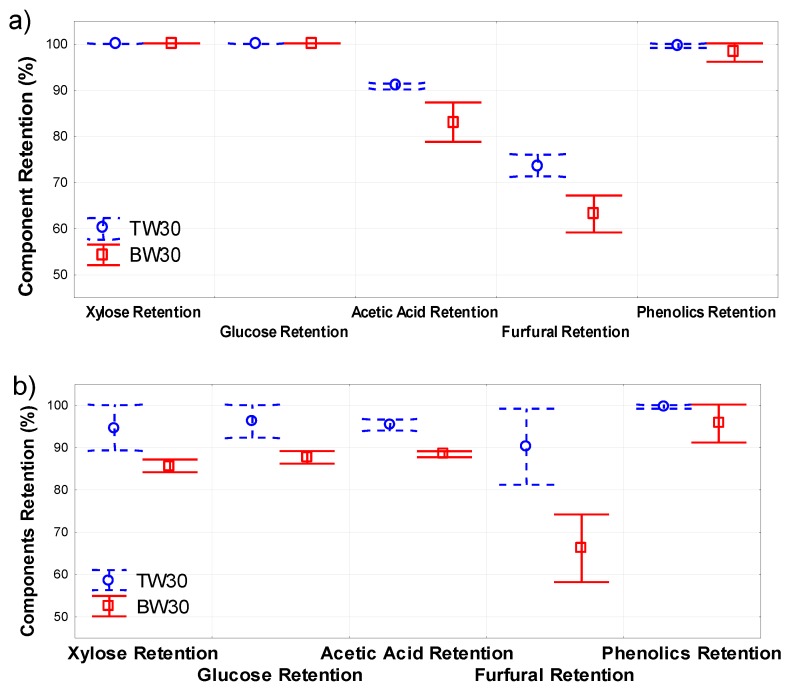
Retention of the prehydrolysate components by membrane at (**a**) minimum, and (**b**) maximum component concentrations.

Two different model solution compositions were used for comparing the separation characteristics of the two membranes (Dow Filmtec TW30 and BW30). The concentration levels were selected to cover the minimum and maximum combination of component concentrations that can be obtained from the levels of furfural concentration, acetic acid, and phenolics for the experimental design given in Table 3. The membrane system was run in a closed loop, to avoid the effect of interference from concentration on the components separation. All screening experiments were carried out at a feed temperature of 36 °C, pressure of 1380 kPa, cross-flow velocity of 0.4 m/s and a duration of 90 min. Samples of the permeate and concentrate were taken at 15 min intervals. Three criteria were used for selecting a membrane. They were the permeate flow, flux decline (fouling potential), and most importantly, the retention of the sugars and acetic acid. The component retention results are shown in Figure 3. All the screening test model solutions contained the same amount of sugars (35 g/L of xylose and 10 g/L of glucose). At the minimum concentration of the other components, the pH and the conductivity of the model solution were 3.55 and 171.8 µS/cm, while they were 2.55 and 904 µS/cm at their maximum concentration. For the screening at the minimum concentration, the retention for xylose, glucose, and phenolics by both membranes was comparable. However, the retention of acetic acid and furfural was slightly higher with the TW30 than the BW30 membrane. At the maximum concentration, the TW30 exhibited higher retention than the BW30 for all components.

The permeate flux for the TW30 membrane was 11% higher than for the BW30 for the minimum concentration solution, but it was 33% less for the maximum concentration solution. The flux decline is indicated by the ratio of pure water flux achieved with the membrane after use for filtration to the pure water flux of the virgin membrane, and it was comparable for both membranes. A decline of 12% was observed for the TW30 membrane, and 13% for the BW30 membrane. The difference in separation characteristics and permeate flux with the two different solutions can be explained as an effect of the interaction between the solution and the membrane. At maximum concentrations, the membranes were prone to flux reduction mechanisms, such as fouling and concentration polarization, which leads to lower permeate flux. The pH and the conductivity of the solution can also influence the zeta potential, and therefore, the observed difference in component separation. Due to the higher component retention with the TW30 membrane, it was selected for use in all the subsequent experiments.

### 4.2. Influence of Variables on Permeate Flux

The ANOVA described in the experimental design section performs a statistical test for determining the most significant factors and their order of significance. Its main goal is to compute the ratio of variation within each factor level to the observed total variation of the results. The computed result consists of several statistical terms: the sum of squares, the mean square (variance), degree of freedom, F-ratio, and *p*-value. The sum of squares reflects the deviation of each run result from the mean of all the results, the degree of freedom is (*n* − 1), where *n* is the number of experiments in a set. The mean square is the ratio of the sum of squares to the degree of freedom. The F-value reveals the magnitude of the influence for each parameter, and it is the ratio of the variance for each parameter divided by the residual variance (error). The *p*-value is the test for significance that shows the probability to obtain the calculated F-values. A parameter is considered to be significant if its *p*-value is less than 0.05. The residue represents the level of uncertainty associated with the experiments. It may represent factors that are beyond control in the experiment design, parameters that were not included in the experiment, or errors while conducting experiments. ANOVA was used to determine which of the six factors has the most significant effect on the permeate flux, the results for the L18 orthogonal arrays are shown in Table 4. The ANOVA results on the impact of the permeate flux can be found in the appendix section (Table A1). The sugar content of the model solutions was the same for all the experiments (35 g/L of xylose and 10 g/L of glucose).

**Figure A1:**
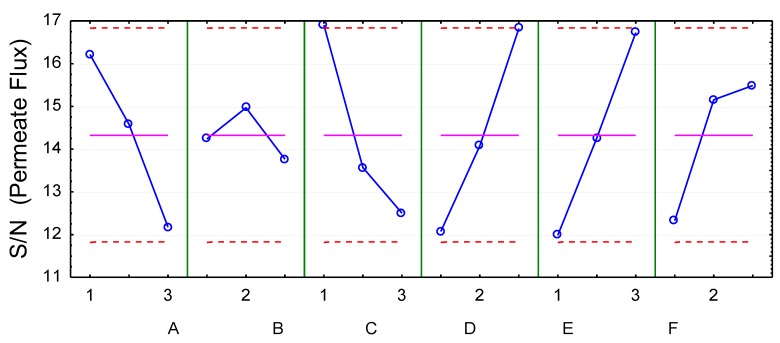
Main effects plot for permeate flux.

The results from the experiments with the TW30 membranes indicate that out of the six parameters studied, the pressure had the highest influence on the permeate flux, followed by the temperature, phenolics concentration, furfural concentration, cross-flow velocity, and acetic acid. Although no single factor exhibited a dominant effect, all the factors contributed to the permeate flux in different degrees, given by the percent factor influence, I. To visualize how the response relates to each of the variables, a graphical representation by the main effects plot is used. It shows the (S/N)_L_ ratios for the experiment parameters (Figure A1). The difference between the highest and the lowest (S/N)_L_ ratio for each parameter indicates the influence of the parameter on the permeate flux.

The larger is better quality characteristic was used to determine the optimum combination of factors. The optimum combination to obtain a high permeate flux is the highest values for each parameter from the main effects plot, and is A1/B2/C1/D3/E3/F3. This optimum combination of variables was not among the L18 treatments in Table 4. Confirmation of the predicted optimum combination gave an initial permeate flux of 95 L/m^2^h, the highest permeate flux obtained in any experimental run.

### 4.3. Influence of Variables on Sugar Retention

**Figure A2:**
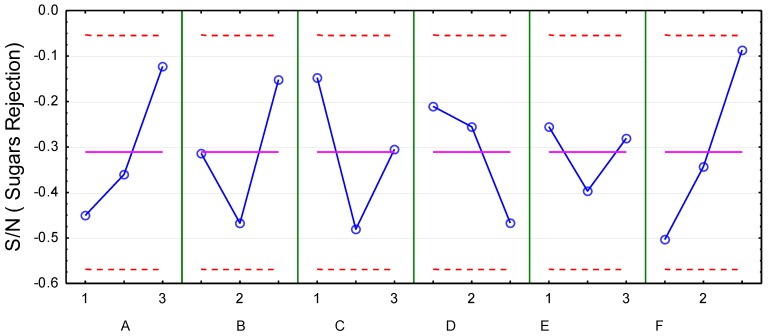
Main effects plot for sugar retention.

ANOVA was also used to evaluate the influence of the variables on the retention of sugars. Two mechanisms (size exclusion and electrostatic repulsion) determine the retention of a specific component during membrane filtration. The average retention of the sugars in all experiments shown in Table 4 was 97%. Results from the analysis of variables given in Table A2 showed that the temperature and pressure had the least influence on the retention of the sugars (where SS: sum of squares; DF: Degree of freedom; MS: Mean sum of squares; F: F-value; p: *p*-value and I (%): the factor influence). It can be considered that rejection of the sugars by the membrane is mainly due to size exclusion, and not to electrostatic repulsion, because the sugars are not charged particles. Increasing the temperature of the feed solution leads to increased membrane pore diameters, and permeation of the solutes and solvent (lower sugars retention). On the other hand, an opposite effect was observed by increasing the concentration of furfural. It can be inferred that the effective pore size of the membrane varies with the concentrations of furfural and the other compounds in the solution, and this could be due to a physical or chemical interaction between the compounds in the solution and the membrane active surface. In addition, the variation can be influenced by the cross-flow velocity. Among the studied variables, the solution composition and the cross velocity (which influences the solute concentrations at the membrane interface) had a more dominant effect on the sugar retention than the temperature and pressure. The optimum points for high total sugar retention are A3/B3/C1/D1/E1/F3, as shown in Figure A2. Nevertheless, the increase in sugar retention that can be achieved using this point is limited, since the average retention observed was 97%.

Further confirmatory experiments were conducted with two model solutions. The sugar compositions, xylose (35 g/L) and glucose (10 g/L), were the same in both solutions. The first solution contained no furfural, acetic acid, and phenols, while the other contained furfural (3.5 g/L), acetic acid (3.5 g/L), and syringaldehyde (2.8 g/L). It was observed that the sugar retention, when only the sugars are present in the model solution, was 100%. However, with the presence of other compounds in the model solution, the retention fell to 97% for xylose, while the glucose retention remained at 100%. Despite the lower molecular weight of xylose compared to glucose (150 vs. 180 g/mol), the observed difference of 3% was not significant.

### 4.4. Influence of Variables on Acetic Acid Retention

**Figure A3:**
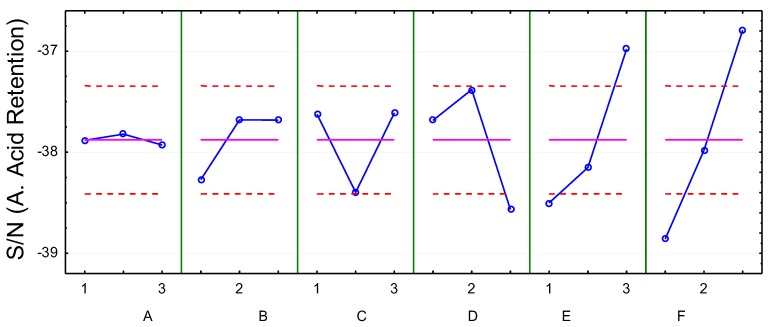
Main effects plot for acetic acid retention.

The analysis of the results has shown that the retention of acetic acid did not depend only on the composition of the model solution. The highest influence came from the cross-flow velocity, followed by the pressure as shown in Table A3. The influences are indicated by SS: sum of squares; DF: Degree of freedom; MS: Mean sum of squares; F: F-value; p: *p*-value and I (%): the factor influence. It can be inferred that since the molecular weight of acetic acid of 68 g/mol is less than the molecular cut off weight (MWCO) of the membrane of 100 g/mol, electrostatic repulsion plays a dominant role in the observed retention. In summary, the retention of acetic acid is strongly affected by any, or a combination of the electrostatic repulsion of the other components and the acetic acid itself. The electrostatic charge interaction at the membrane surface depends on the pH of the solution, which is also determined by the degree of ionization of the acid, the membrane characteristics, and the operating conditions. Acetic acid retention was between 61% and 91% as shown in Table 4. The optimum point for high acetic acid retention is A2/B2/C3/D2/E3/F3, and it is illustrated in Figure A3. Confirmatory experiments at the optimum condition showed that 92% retention of acetic acid can be achieved. This does not differ significantly from the highest value obtained in the experiment design, which was 91%. 

Further experiments to expand the observed trend were carried out with two different model solutions. The first solution contained only acetic acid, while the second had all the other components, furfural (3.5 g/L), acetic acid (3.5 g/L), and syringaldehyde (2.8 g/L), in addition to sugars. The acetic acid retention in the mixture was 74%, and 51% for the solution containing only acetic acid. A more complex mixture resulted in higher acetic acid retention. This cannot be explained by electrostatic charge interaction, because both solutions have a similar pH of about 3, and no significant difference between the solutions and membrane active surface is expected. Verliefde et al. have also shown that the retention of organic acids is not driven by charge interactions only, and steric interactions also play a significant role [54]. Furthermore, Teella et al. carried out binary solution filtration experiments and showed that the retention of acetic acid significantly decreases in the presence of glucose [20]. Hence, the observation could be explained by the presence of other compounds, which alter the retention characteristics or the increased concentration polarization, due to the higher flux in the solution containing only acetic acid. 

### 4.5. Influence of Variables on Flux Decline

**Figure 4:**
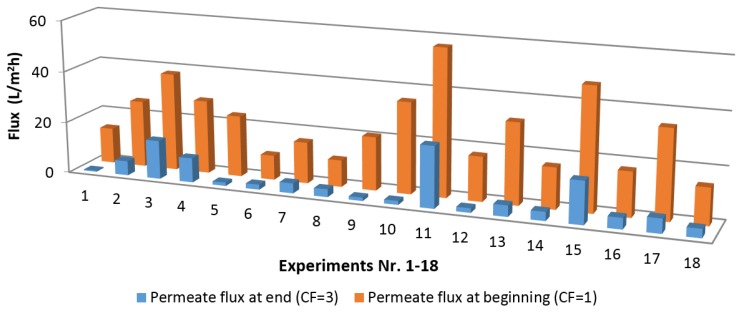
Initial and final permeate flux for the L18 Taguchi experiments.

The C5 sugar concentration has to be increased from 35 g/L to at least 105 g/L (a concentration factor of 3) to obtain a furfural composition comparable to most reported process, during the subsequent conversion step. Comparison of the permeate flow at the beginning of concentration and end of concentration was carried out, and is shown in Figure 4. The flux at the beginning and end of each experiment depends on the model solution composition and operating conditions shown in Table 4. A permeate flux decline was observed during all the concentration experiment runs. Some of the experiments with the highest initial flux (experiments 3, 11 and 15) were carried at the highest feed pressure. Although the concentration factor for all the 18 model solutions was 3, the flux decline was greater than a factor of 3 in about 77% of the experiments. This indicates the presence of other flux decline mechanisms, different from the osmotic pressure increase. Furthermore, the permeate flux observed in half of the experiments was lower than a typical heuristic design permeate flux (21 L/m^2^h) that corresponds to 5 mL/min in Figure 4.

The influence of the six factors determined by the analysis of variables is given in the appendix section (Table A4). The order of ranking is cross-flow velocity > pressure > temperature > phenolics > acetic acid > furfural. The results imply that an interaction between the physical parameters and the model solution components was responsible for the flux decline. In addition, compaction can be excluded from the main flux decline mechanisms, because the preliminary characterization tests described in Section 2.4 did not reveal compaction of the membrane for the selected cross-flow velocity, pressure and temperature.

As a result of the analysis, five sets of confirmatory tests were performed to show the individual effects of each model solution component. All experiments contained different amounts of the constituent compounds, as shown in Table 5. The selected feed conditions were 40 °C, 3100 kPa, and a cross velocity of 0.4 m/s. The same initial sugars concentration for the 18 experiments was also used in the confirmation experiments. 

**Figure 5:**
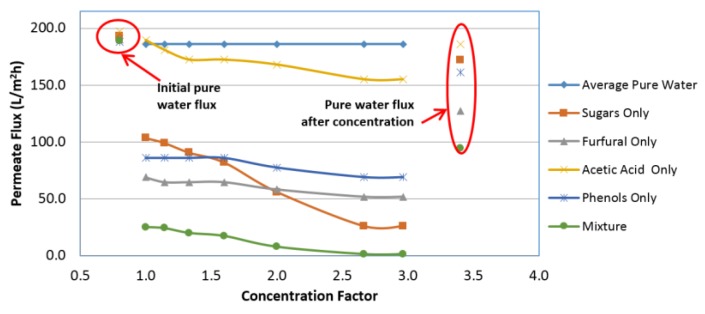
Comparison of flux decline caused by the model solution components.

The results allowed for identification of different flux decline mechanisms. As shown in Figure 5, the measured initial permeate flux was approximately 194 L/m^2^h for all experiments. Concentration by withdrawal of the permeate stream commenced 3 min after the feed pump was started, thus corresponding to a concentration factor of 1.0. The lowest permeate flux decline was observed for the model solution containing only acetic acid. A pure water flux of 185 L/m^2^h was achieved after this concentration run and flushing of the system with distilled water. This indicated that the presence of acetic acid in the model solution did not contribute to the flux decline observed. The permeate flux for the sugar solution fell from 103 L/m^2^h at the onset to about 26 L/m^2^h after concentration. The ratio of initial to the final permeate flow was 4, and it can be concluded that since the ratio is in the same range as the concentration factor which is 3, the reduction experienced with the sugar only solution can be attributed to the osmotic pressure, which is directly proportional to the concentration factor. The difference in permeate flux between the beginning and end of concentration was less than 21 L/m^2^h for the three other model solutions: furfural only, phenols only, and mixture. It can also be seen that the decline with these three solutions was present from the onset of concentration. 

**Figure 6:**
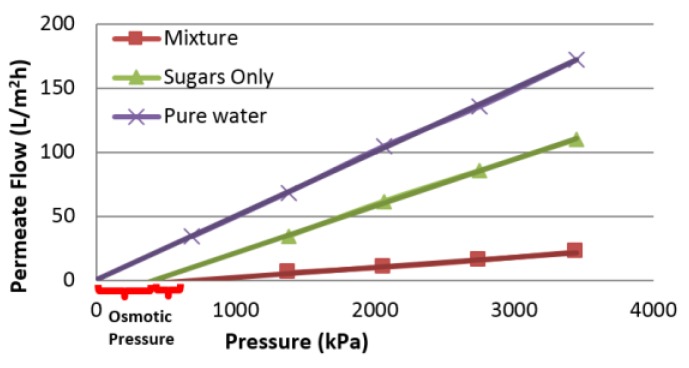
Effect of model solution components on the apparent osmotic pressure.

A comparison of the pure water flux obtained after each concentration experiment revealed that the model solutions containing furfural resulted in the highest pure water flux decline. The flux reduction by the phenolic compound is less than that of furfural. Experiments to determine the osmotic pressure contribution of each component with the same composition, shown in Table 5, was also performed. Results showed that the osmotic pressure in the acetic acid only, phenols only, as well as furfural only solution, tends to zero. The effect of the model solution component on the osmotic pressure using the same graphical approach is shown in Figure 6.

**Figure 7:**
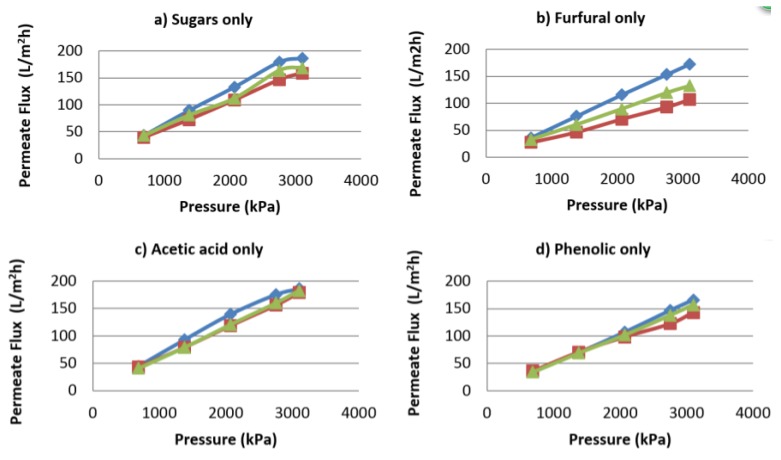

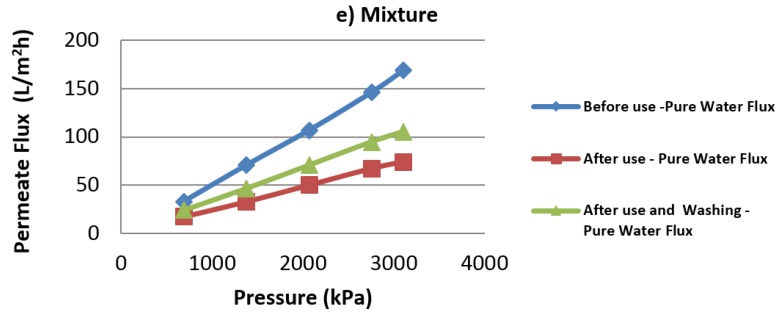
Permeate flow against feed pressure to differentiate fouling from concentration polarization.

The difference between each intercept and the pure water intercept is an approximation of the order of magnitude of the osmotic pressure. The graphical extrapolation is based on the premise that there is no concentration polarization present. To ensure this was valid, a series of runs in which the cross-flow velocity varied between 0.3 and 0.5 m/s were carried out to confirm that no significant concentration polarization was present. As a result, chemical cleaning (NaOH solution) also did not result in increased flux. It became evident that the presence of furfural contributes to the flux decline of the membrane by fouling. This can be seen in Figure 7b,e for which the highest flux decline was observed.

The difference in osmotic pressure between the sugar only and the mixture solutions can be explained by the occurrence of flux decline mechanisms. It can thus be inferred that the flux decline caused by the presence of sugars is due to the osmotic pressure, while for furfural and the phenolic compound, it can be due to either fouling of the membrane, concentration polarization, or both. The flux decline experienced due to each of the model solution components can be classified as either physically or chemically reversible. Physical reversibility refers to the fraction of the initial pure water flux that can be obtained after the membrane has been used and subsequently cleaned with distilled water. Chemically reversibility is the fraction that can be obtained after use and subsequent cleaning with a sodium hydroxide solution, a base cleaning agent. The effectiveness of using a cleaning agent can also be seen. This suggests that a sodium hydroxide based cleaning agent can partially reverse the fouling caused by furfural. Since fouling of membrane cannot be completely eliminated, being able to regenerate a membrane by cleaning is an essential factor to be considered, to make membrane concentration feasible. The fouling tendency of furfural was confirmed by visual inspection. A discolored fouled area was observed on the membrane surface for all experiments in which furfural was present in the model solutions. This area was similar for a solution containing only furfural.

### 4.6. Comparison with Filtration of Real Prehydrolysate Solution

To be able to compare the performance of the membrane for a model solution run to that of a real prehydrolysate, a separate experimental run was performed. The real solution described in Section 2.4, and the optimum filtration conditions (30 °C, 2800 kPa, and 0.45 m/s) for the trial were determined from a previous study [28]. It was observed that the component retention was in the same range as that of the model solution shown in Table 2, with sugars at 99% and acetic acid at 89%, respectively. However, a difference was seen for the permeate flux. The initial permeate flux at 31 L/m^2^h was significantly lower than the 95 L/m^2^h for the model solution under optimum conditions and composition. The final permeate flux was 8.4 L/m^2^h after filtration was stopped. Subsequent filtration with pure water showed that after the real prehydrolysate run, the pure water flux reduced by 29%. However, after washing of the membrane, the reduction was only 16%.

## 5. Conclusions

The cumulative impact that the main chemical compounds found in prehydrolysate solutions from a dissolving pulp mill have on RO membrane filtration was studied using model solutions containing glucose, xylose, acetic acid, furfural, and syringaldehyde. Two commercial membranes, BW30 and TW30, were evaluated, and the TW30 membrane was shown to be the most efficient for the simultaneous concentration of the sugars and acetic acid. It is important to retain the acetic acid, because it serves as a catalyst for the conversion of pentoses into furfural. The dependence of the component retention on the composition and operating conditions was determined. The Taguchi design of experiments method led to the identification of furfural concentration as a significant cause of permeate flux decline. Further experiments led to the identification of decline mechanisms caused by the key compounds that can be found in prehydrolysate solutions. It is expected that since membrane cleaning after use with a solution containing only sodium hydroxide was possible, the use of commercial cleaning agents will also be possible for regenerating the membrane. The experimental results suggest that RO membrane filtration is technically feasible, and can be successfully applied to the concentration of hemicellulose prehydrolysate in a furfural production process. Furthermore, the chemical composition of prehydrolysate solution is a factor that cannot be ignored when concentrating sugars from prehydrolysate solutions. The constituent chemical compounds have a synergistic impact on the retention and flux decline characteristics. 

## Figures and Tables

**Table 1:** Previous works on hydrolysate treatment using filtration reverse osmosis (RO), nanofiltration (NF), and ultrafiltration (UF) membranes.

Memb. Type	Prehydrolysate Type (Compounds Present)	Objectives	Key Results/Observations	Ref.
NF	Synthetic solution: (xylose, glucose, furfural)	Sugar purification for ethanol	Concentration and purification can be accomplished	[15]
UF	Corn waste hydrolysis liquor: (glucose, xylose, arabinose, and acetic acid)	Hydrolysate purification bioproducts	Ultrafiltration can be used for hemicellulose fractionation and purification	[16]
NF	Hemicellulosic hydrolysate	Inhibitor removal for butanol	Removal of nearly all small molecular organic acids, furfural, and HMF is possible	[17]
NF	Rice straw hydrolysate (glucose, xylose, arabinose, cellobiose, fructose, sucrose, acetic acid, HMF, rurfural, ferulic acid, vanilic acid)	Inhibitor removal	Simultaneous concentration of sugars and separation of inhibitors achievable	[21]
NF	Three sugar solution (glucose solution, diluted sugar beet molasses, and liquid hydrolysate of dilute acid-pretreated rice straw, glucose, xylose, acetate formate, furfural, and HMF)	Sugar concentration and inhibitor removal	Sugars can be concentrated and fermentation inhibitors removed at low pressures prior to successful fermentation	[22]
RO & NF	Hydrothermal iquefaction (HTL) hydrolysates (glucose, xylose, acetic acid, lactic acid, levulinic acid, phenol, 2-methoxyphenol, and 2,6-dimethoxyphenol)	Hydrolysate fractionation	Two-stage membrane process is feasible for fractionating model HTL hydrolysates	[23]
RO & NF	Lignocellulosic hydrolysate model solution (C5 and C6 sugars from acetic acid, furfural, 5-hydroxymethyl furfural, and vanillin in a model solution)	Inhibitor removal	RO had the highest sugar retention but inhibitor removal was lower than for NF	[24]
RO & NF	Hemicelluloses prehydrolysate (glucose, mannose, galactose, xylose, arabinose, acetic acid, furfural)	Inhibitor removal	Membrane filtration not efficient for phenolic inhibitors removal except in combination with flocculation	[25,26]
RO & NF	Corn stover hydrolysate (glucose, xylose, acetic acid, furfural, and HMF) Model solution (glucose, xylose, acetic acid, furfural, and HMF)	Inhibitor removal	Hydrolysis degradation products can be removed, but membrane surface characteristics play a role	[27]
RO & NF	Hemicellulose prehydrolysate (glucose, mannose, galactose, xylose, arabinose, acetic acid, furfural)	Sugar and inhibitor concentration	Retention and flux characteristics determined, but no indication of the impact of components	[28]
NF	Model solution (vanillic acid, *p*-coumaric acid, ferulic acid, xylose, arabinose, and glucose)	Phenolics removal	Enzymes can be used to polymerize phenolic compounds and facilitate their separation from sugars	[29]
RO & NF	Lignocellulosic hydrolysate mix (glucose, xylose, mannose, galactose, and arabinose, furfural, HMF, acetic, and other unidentified organic acids) Synthetic pure sugar mix (glucose, xylose, mannose, galactose, and arabinose)	Sugar concentration and inhibitor removal	Higher inhibitor separation comes with sugar losses, and reversible fouling was mainly responsible for flux reduction	[30]
UF & MF	Rice straw hydrolysate (sugar mix indicated by reducing sugars)	Sugar recovery and inhibitor removal	The effects of membrane type, pore size, cross-flow velocity, and transmembrane pressure on the filtration flux, and sugar rejection elucidated	[31]
NF	Wheat straw pretreatment liquor (mono and oligosaccharides, acids and furans) Model solutions (sodium chloride, potassium chloride, acetic acid, formic acid, 5-hydroxymethylfurfural (HMF), d-xylose, l-arabinose, d-glucose, and xylan)	Acid and furan removal	Diananofiltration strategy shown to be promising for the recovery of high-purity streams of monosaccharides	[32]
RO, NF & UF	Spruce wood autohydrolysate	Recovery of hemicelluloses	Diafiltration and pulsed corona discharge (PCD) improves recovery	[33]
UF	Wheat bran hemicelluloses (araboxylan) solution	Concentration and purification	Product purity and ultrafiltration performance can be improved by dead-end prefiltration	[34]
NF & UF	Pine wood autohydrolysis liquor from containing poly- and oligosaccharides (POHS), and monosaccharides	Concentration, purification, and fractionation	The purified POHS/monosaccharides ratio can be altered by different membrane combinations	[35]
UF	Birch chips and spruce saw-dust hydrolysate	Fouling reduction while removing inhibitors	Pulsed corona discharge (PCD) and activated carbon treatments reduces fouling	[36]

**Table 2:** Physicochemical properties of the model solution compounds used in this study.

Chemical	d-Glucose	d-Xylose	Acetic Acid	Syringaldehyde	Furfural
Formula	C_6_H_12_O_6_	C_5_H_10_O_5_	C_2_H_4_O_2_	C_9_H_10_O_4_	C_5_H_4_O_2_
Molecular Structure	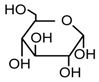	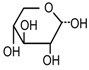	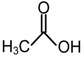	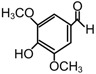	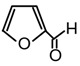
MW (g/mol)	180.16	150.13	60.05	182.17	96.08
D (×10^−5^ cm^2^/s)	0.67 [40]	0.75 [40]	1.29 [41]	n/a	1.01 [42]
p*K*_a_	12.46 [41]	12.14 [41]	4.76 [41]	7.34 [43]	n/a

MW: molecular weight; D = diffusion coefficient at 25°C; and p*K*_a_: dissociation constant.

**Table 3:** Experimental design of six controlling factors with three levels.

Controlling Factors	Levels			Units
	1	2	3	
A (Furfural concentration)	0.6	1.8	3.5	g/L
B (Acetic acid concentration)	0.5	3.5	10	g/L
C (Phenolics concentration)	0.3	2.8	6	g/L
D (Temperature)	20	30	40	°C
E (Pressure)	3100	3800	4500	kPa
F (Cross-flow velocity)	0.3	0.4	0.5	m/s

**Table 4:** Orthogonal array of L18 (3^6^) and measured parameters.

Exp Nrs.	Levels of parameters						R_S_	R_A_	J_P-i_	J_P-i_/J_P-f_	pH
	A	B	C	D	E	F	(%)	(%)	(L/m^2^h)		
1	1	1	1	1	1	1	0.95	0.74	14	32	3.65
2	1	2	2	2	2	2	0.92	0.70	26	5	3.25
3	1	3	3	3	3	3	1.00	0.78	38	3	2.68
4	2	1	1	2	2	3	1.00	0.83	28	3	3.25
5	2	2	2	3	3	1	0.86	0.88	24	18	2.67
6	2	3	3	1	1	2	0.98	0.81	9	4	2.69
7	3	1	2	1	3	2	0.99	0.81	16	4	3.23
8	3	2	3	2	1	3	0.98	0.79	10	3	2.94
09	3	3	1	3	2	1	0.97	0.61	21	16	2.70
10	1	1	3	3	2	2	0.90	0.62	35	21	3.28
11	1	2	1	1	3	3	0.99	0.79	57	2	3.36
12	1	3	2	2	1	1	0.95	0.66	17	10	2.66
13	2	1	2	3	1	3	0.97	0.71	31	7	3.06
14	2	2	3	1	2	1	0.96	0.91	16	5	2.79
15	2	3	1	2	3	2	1.00	0.79	47	3	2.61
16	3	1	3	2	3	1	0.98	0.84	17	4	3.20
17	3	2	1	3	1	2	0.99	0.70	34	6	2.85
18	3	3	2	1	2	3	1.00	0.91	14	4	2.66

**Table 5:** Model solution composition for the confirmation of permeate flux decline.

Expt Nr.	Description	A (g/L)	B (g/L)	C (g/L)	Sugars	
					X (g/L)	G (g/L)
1	Sugars only	0	0	0	35	10
2	Acetic acid only	3.5	0	0	0	0
3	Furfural only	0	3.5	0	0	0
4	Phenols only	0	0	2.8	0	0
5	Mixture	3.5	3.5	2.8	35	10

A: furfural concentration; B: acetic acid concentration; C: phenolics concentration; X: xylose concentration; G: glucose concentration.

## Appendix A

The figures and tables for the analysis of variance (ANOVA) results obtained for the impact of parameters on permeate, on retention of sugars, acetic acid retention as well as the parameters influencing flux reduction are provided in this section.

**Table A1:** ANOVA tables for impact of operating parameters on permeate flux.

		SS	DF	MS	F	p	I (%)
1	A (Furfural concentration)	35.7	2	17.9	1.4	0.3	10.8
2	B (Acetic acid concentration)	5.2	2	2.6	0.2	0.8	1.6
3	C (Phenolics concentration)	51.8	2	25.9	2.1	0.2	15.6
4	D (Temperature)	67.7	2	33.9	2.7	0.2	20.4
5	E (Pressure)	85.6	2	42.8	3.5	0.1	25.8
6	F (Cross-flow velocity)	23.5	2	11.7	0.9	0.4	7.1
	**Residue**	**61.9**	**5**	**12.4**			**18.7**

SS: sum of squares; DF: Degree of freedom; MS: Mean sum of squares; F: F-value; p: *p*-value and I (%): the factor influence.

**Table A2:** ANOVA tables for impact of operating parameters on retention of sugars.

		SS	DF	MS	F	p	I (%)
1	A (Furfural concentration)	0.3	2	0.2	1.7	0.3	15
2	B (Acetic acid concentration)	0.3	2	0.1	1.5	0.3	13
3	C (Phenolics concentration)	0.3	2	0.2	1.7	0.3	14.5
4	D (Temperature)	0.2	2	0.1	1.1	0.4	9.8
5	E (Pressure)	0.1	2	0	0.3	0.7	3
6	F (Cross-flow velocity)	0.5	2	0.3	2.7	0.2	23.1
	**Residue**	**0.5**	**5**	**0.1**			**21.7**

SS: sum of squares; DF: Degree of freedom; MS: Mean sum of squares; F: F-value; p: *p*-value and I (%): the factor influence.

**Table A3:** ANOVA tables for impact of operating parameters on acetic acid retention.

		SS	DF	MS	F	P	I (%)
1	A (Furfural concentration)	0.03	2	0.02	0.04	0.96	0.1
2	B (Acetic acid concentration)	1.40	2	0.70	1.61	0.29	4.7
3	C (Phenolics concentration)	2.4	2	1.22	2.82	0.15	8.3
4	D (Temperature)	4.6	2	2.27	5.28	0.06	15.5
5	E (Pressure)	7.6	2	3.81	8.84	0.02	25.9
6	F (Cross-flow velocity)	12.9	2	06.45	14.94	0.01	43.8
	**Residue**	**2**	**5**	**0.43**			**1.7**

SS: sum of squares; DF: Degree of freedom; MS: Mean sum of squares; F: F-value; p: *p*-value and I (%): the factor influence.

**Table A4:** ANOVA tables for the parameters influencing flux reduction.

		SS	DF	MS	F	P	I (%)
1	A (Furfural concentration)	0.04	2	0.02	0.04	0.96	0.1
2	B (Acetic acid concentration)	1.39	2	0.70	1.62	0.29	4.5
3	C (Phenolics concentration)	2.43	2	1.22	2.82	0.15	7.8
4	D (Temperature)	4.55	2	2.28	5.28	0.06	14.6
5	E (Pressure)	7.62	2	3.81	8.84	0.02	24.5
6	F (Cross-flow velocity)	12.89	2	6.45	14.95	0.01	41.5
	**Residue**	2.16	5	0.43			6.9

SS: sum of squares; DF: Degree of freedom; MS: Mean sum of squares; F: F-value; p: *p*-value and I (%): the factor influence.
